# The Role of the Multiple Hormonal Dysregulation in the Onset of “Anemia of Aging”: Focus on Testosterone, IGF-1, and Thyroid Hormones

**DOI:** 10.1155/2015/292574

**Published:** 2015-12-08

**Authors:** Marcello Maggio, Francesca De Vita, Alberto Fisichella, Fulvio Lauretani, Andrea Ticinesi, Graziano Ceresini, Anne Cappola, Luigi Ferrucci, Gian Paolo Ceda

**Affiliations:** ^1^Department of Clinical and Experimental Medicine, Section of Geriatrics, University of Parma, 43126 Parma, Italy; ^2^Geriatric Rehabilitation Department, University Hospital of Parma, 43126 Parma, Italy; ^3^Division of Endocrinology, Diabetes, and Metabolism, Department of Medicine, Perelman School of Medicine at the University of Pennsylvania, Philadelphia, PA 19104, USA; ^4^National Institute on Aging, National Institutes of Health (NIH), Baltimore, MD 21201, USA

## Abstract

Anemia is a multifactorial condition whose prevalence increases in both sexes after the fifth decade of life. It is a highly represented phenomenon in older adults and in one-third of cases is “unexplained.” Ageing process is also characterized by a “multiple hormonal dysregulation” with disruption in gonadal, adrenal, and somatotropic axes. Experimental studies suggest that anabolic hormones such as testosterone, IGF-1, and thyroid hormones are able to increase erythroid mass, erythropoietin synthesis, and iron bioavailability, underlining a potential role of multiple hormonal changes in the anemia of aging. Epidemiological data more consistently support an association between lower testosterone and anemia in adult-older individuals. Low IGF-1 has been especially associated with anemia in the pediatric population and in a wide range of disorders. There is also evidence of an association between thyroid hormones and abnormalities in hematological parameters under overt thyroid and euthyroid conditions, with limited data on subclinical statuses. Although RCTs have shown beneficial effects, stronger for testosterone and the GH-IGF-1 axis and less evident for thyroid hormones, in improving different hematological parameters, there is no clear evidence for the usefulness of hormonal treatment in improving anemia in older subjects. Thus, more clinical and research efforts are needed to investigate the hormonal contribution to anemia in the older individuals.

## 1. Introduction

### 1.1. Anemia and Unexplained Anemia in the Elderly

Anemia is a multifactorial condition whose prevalence increases in both sexes after the fifth decade of life, becoming a highly represented phenomenon in older adults [1]. The estimated prevalence of anemia highly varies according to the study population, study setting, and health status, as well as the criteria to define anemia [2]. The diagnosis is more frequently based on the World Health Organization (WHO) criteria reported in 1968, as hemoglobin (Hb) concentration <12 g/dL and <13 g/dL in nonpregnant women and men, respectively [3]. Data from the Third National Health and Nutrition Examination Survey (NHANES III), by using the WHO definition, have estimated a prevalence of anemia of about 11% of community-dwelling older men and 10.2% of women, exceeding 40% among the oldest-old population (85 years and older) [4]. Interestingly, the prevalence of anemia is greater among nursing home residents than in community-dwelling older individuals [2, 5, 6].

In the elderly, anemia is caused by different factors including nutrient deficiencies (iron, folate, and vitamin B12), occult hemorrhage, renal dysfunction, and chronic inflammation/diseases [7]. However, data report that in about one-third of anemic older adults the determinants of this condition cannot be clearly identified and anemia is defined as “unexplained” (UA) [8]. Anemia “sine causa” is typically mild (with Hb levels approximately 1 g/dL lower than the WHO standard), featured by normal red blood cells size with no evidence for intravascular destruction or morphological features [7]. All these characteristics make this condition not frequently recorded up to 75% of the cases [9].

Although mild anemia can be well tolerated in young and adult individuals, this condition in the elderly is a predictor of a progressive decrease in physical performance and muscle strength [10, 11], and an increased risk of recurrent falls [12], frailty status [13], cognitive impairment [14], and hospitalization [15, 16].

Moreover, large population-based prospective studies performed in community-dwelling older individuals identified anemia as risk factor for death, independently of multimorbidity [17, 18]. An association between mild anemia and multiple adverse outcomes has also been shown among vulnerable class of individuals, such as those with chronic heart failure [19], chronic kidney disease [20], and diabetes mellitus [21]. However, it is still unclear whether anemia has a causative role in the development of adverse events or is just a marker of the disease burden [22].

### 1.2. Contributing Factors of UA in the Elderly: The Role of “Multiple Hormonal Dysregulation”

Efforts to understand the pathophysiology of the so-called anemia of aging have specifically targeted different physiological age-related changes including erythropoietin (EPO) resistance and reduced proliferative capacity of bone marrow stem cells, stem cell aging, impaired renal function, myelodysplasia (MDS), and chronic inflammation with higher circulating levels of proinflammatory cytokines [8, 23, 24]. These contributing factors might lead to alterations in the red blood cell production and red blood cell survival.

Among the predisposing factors of anemia recent studies underline the role of hepcidin, an iron-regulating hormone, which at higher levels seems to favour both iron sequestration in reticuloendothelial cells and the alteration of gut ferroportin-mediated iron absorption [25]. However, the role of hepcidin in the onset of UA is still controversial [26].

**Figure 1:**
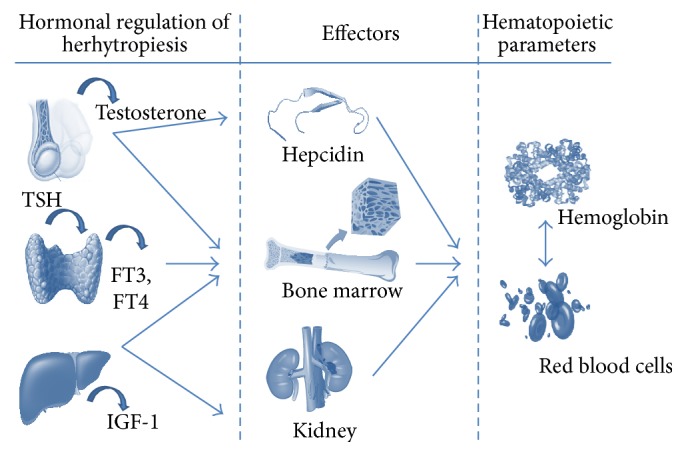
Potential molecular mechanisms underlying the hormonal regulation of erythropoiesis. The figure depicts the potential molecular mechanisms underlying the hormonal regulation of erythropoiesis (IGF-1, T, and thyroid hormones) hypothesized in both* in vitro* and animal studies.* Testosterone*. By binding with a nuclear androgen receptor (AR), testosterone may exert a direct role on bone marrow erythroblast and red cell precursor survival leading to an increase in erythroid mass and erythroid and myeloid colony formation. The erythropoietic activities of T may also be related to the stimulation of EPO synthesis and secretion at kidney level (by modulating the hypoxia or hypoxic sensing). Testosterone also enhances the sensitivity of erythroid progenitor cells to EPO, determining an increase in red cell production. An alternative hypothesis considers the suppression of hepcidin, the iron-regulating hormone, as a contributory factor in the T-related erythrocytosis. Testosterone might reduce hepcidin levels by modulating inflammatory cytokines, especially interleukin-6 (IL-6), known modulator of the liver production of hepcidin.* IGF-1*. IGF-1 directly stimulates the proliferation and differentiation of the late stage of primitive erythroid progenitor cells and/or early erythroid progenitor cells. IGF-1 might also increase the expression of cell-surface transferrin-receptors by determining a redistribution from the intracellular compartment to the cell surface. Alternatively, IGF-1 can act together with EPO in a synergistic way suggesting for IGF-1 a role of EPO substitute.* TSH and Thyroid Hormones*. Thyroid-stimulating hormone (TSH), L-triiodothyronine (T3), and L-thyroxine (T4) might play a direct role in ensuring normal erythropoiesis. Thyroid-stimulating hormone could affect hematopoiesis by binding to a functional thyrotropin receptor (TSHR), which is found in both erythrocytes and some extrathyroidal tissues. T3 is involved in the control of growth and apoptosis of hematopoietic cells and bone marrow tissue by potentiating the erythroid burst-forming unit (BFU-E) proliferation. T4 has been shown to exert a direct, *β*2-adrenergic receptor-mediated stimulation of red cell precursors. The effects of thyroid hormones on erythropoiesis seem to be mediated at the molecular level by the T3 binding to specific nuclear receptors (the endogenous receptor alpha, c-erbA/TR*α*) and the closely related retinoic acid receptor *α* (RAR*α*) involved in the regulation of normal erythroid differentiation. In hypothyroid conditions, reduced EPO levels might also account for anemia.

Interestingly, the age-related decline in androgen levels has been recently numbered among the factors involved in the pathogenesis of the so-called UA of the elderly [27, 28]. Epidemiological studies and several clinical trials have suggested a potential relationship between Hb levels and the severity of androgen deficiency [29]. Serum insulin-like growth factor 1 (IGF-1) and insulin-like growth factor-binding protein 3 (IGFBP-3), thyroid-stimulating hormone (TSH), L-triiodothyronine (T3), and L-thyroxine (T4) levels seem also to be involved in maintaining Hb concentration under physiological and pathological conditions (Figure 1).

Therefore, anabolic hormones, whose levels undergo a progressive decline during ageing, might represent a plausible cause of anemia when other causal pathways have been excluded. Interestingly, the ageing model is frequently characterized by a simultaneous deficiency in anabolic hormones. Multiple hormonal “dysregulation” is a more frequent phenomenon than a single hormonal derangement in older persons [30]. This phenomenon mostly involves gonadal, adrenal, and somatotropic axes, with a subsequent decline in testosterone (T), dehydroepiandrosterone sulfate (DHEA-S), and IGF-1 concentrations, respectively [30], resulting in anabolic deficiency, an important determinant of accelerated aging process [30]. There is recent evidence that anabolic hormones do not operate independently of each other and might have synergistic effects [30]. It is very well known that T mainly acts as anabolic agent while GH exerts an anticatabolic affect [31]. IGF-1 can be also considered the cross-road of many stimuli including DHEAS and T [30]. Data from Hagenfeldt and colleagues [32] suggest that androgen therapy is able to increase IGF-1 levels, which may represent an important mediator of T action.

A multiple hormonal derangement, rather than a single anabolic hormone deficiency, has been identified as a robust biomarker of health status in older persons [33]. Data from the Aging in the Chianti Area (InCHIANTI) Study documented that the parallel decline in T, DHEAS, and IGF-1 independently predicts 6-year mortality in older men [33].

According to this concept a partial failure of a single hormone system might be compensated by one or more parallel systems without producing significant detrimental clinical effects, suggesting that the single hormonal derangement is a not specific predictive parameter in older individuals.

Data were especially focused on the erythropoietic properties of a single anabolic agent (T, IGF-1, thyroid hormones, or estradiol), with limited evidence existing on the contribution of multiple anabolic pathways in the pathogenesis of anemia.

Thus, we performed an accurate review of the literature using PubMed until March 2015, by selecting the observational human studies investigating the hormonal contribution (Testosterone, IGF-1, and thyroid Hormones) to anemia older subjects of both sexes. In this review, studies are presented according to the cross-sectional and longitudinal design and to the positive or neutral effects on different hematological parameters. We also analyzed the clinical trials having hematological indexes as the main or secondary outcome.

## 2. Testosterone and Anemia

### 2.1. Potential Mechanisms

The role of androgens on erythropoiesis is known since 1941 [34] when, before the availability of recombinant human EPO, they represented the main pharmacologic agents in the treatment of anemia of chronic and end-stage renal disease, as well as aplastic anemia [35, 36].

*In vitro* and animal studies have suggested a role for androgens in increasing erythroid mass, EPO synthesis, and iron bioavailability. In bone marrow cultures, androgens stimulate hematopoiesis [37–39] also improving erythroid and myeloid colony formation in semisolid cultures [40, 41]. However, myelostimulating effects of androgens seem to target more mature erythroid progenitors rather than hematopoietic stem cells or immature progenitor cells [42]. In human cell cultures, T exerts a direct role on bone marrow erythroblasts and red cell precursor survival [28, 43, 44], by binding with a nuclear androgen receptor (AR) [45]. Interestingly, the pretreatment of bone marrow cells with cyproterone and flutamide, selective competitive blockers of nuclear AR [46], completely inhibits the beneficial effects of T on erythroid burst-forming units and colony-forming units. In mice, T administration increases Hb levels and hematocrit as well as granulocyte and platelet numbers, with a greater effect in the oldest experimental study group [47, 48].

Most of the erythropoietic activities of T seem to be mediated by the stimulation of EPO secretion [49–51] and by the modulation of erythroid progenitor cells sensitivity to EPO, overall resulting in an augment of the red cells production.* In vitro* studies have documented stimulatory properties for androgens on red cell synthesis, blocked by injection of anti-EPO antibody [52]. Furthermore, erythroid progenitor cells from mice treated with T exhibited an increased proliferative response to EPO [37, 46]. However, a modest increase in EPO concentrations has been also observed in patients who underwent total androgen blockade therapy (short-term and long-term), after the decline in serum Hb levels, independently of serum T concentration [53]. There is evidence that T increases EPO levels by modulating the hypoxia or hypoxic sensing, known to stimulate EPO secretion [54]. Therefore, many effects induced by T (including increased Hb and hematocrit, increased red cell 2,3-bisphosphoglycerate, and increased muscle capillarity) increase the net oxygen delivery to the tissue [55]. Alternatively, T could influence EPO secretion via direct effects at renal peritubular fibroblasts level [56], establishing a new EPO/Hb “set point.” A similar phenomenon is observed in post-transplant erythrocytosis, renal dysfunction, and some populations who live at high altitude [57]. In contrast, other data describe a direct action of T in hypoxia-induced EPO synthesis. These studies show no effect of T on EPO transcription in Hep3B cells, an EPO-secreting cell line highly sensitive to hypoxic stimuli [58].

However, mechanisms other than EPO have been proposed to explain the role of T deficiency in the decline of both Hb and hematocrit levels. Animal [59, 60] and human [61] studies reported that T may influence iron bioavailability and utilization.

Guo and colleagues [55] recently argued that the increased transferrin levels and reduced serum ferritin concentration could explain the increased Hb concentration observed after T administration. However, T replacement therapy with T propionate seems more effective in stimulating erythropoiesis when chronic administration is established [62].

It is very well known that serum transferrin concentration reflects the body iron status and erythroid transferrin uptake as well as total erythroid activity [63]. An alternative hypothesis suggests a role for the suppression of hepcidin, the iron-regulating hormone factor, in the T-related erythrocytosis. Testosterone might reduce hepcidin levels by decreasing inflammatory cytokines, especially interleukin-6 (IL-6) [64]. Elevated levels of IL-6 are known to negatively influence erythropoiesis [64], also increasing the liver production of hepcidin through the HAMP gene [7, 65, 66]. It is very well documented that T exerts anti-inflammatory properties by inhibiting the proinflammatory nuclear transcription factor kappa B (NF*κ*B) [67] and the expression of the inflammatory mediator IL-6. Bone marrow cell cultures treated with serum from patients affected by chronic disease exhibited a suppression of erythroid colony-forming units (CFU-E) [68]. Interestingly, this effect reversed after the administration of antibodies against TNF*α* and IFN*γ* [68]. In erythroid cells treated with T, an increase in both iron export from the spleen and iron availability for Hb synthesis has been observed as consequence of the suppression of hepcidin and the upregulation of ferroportin (the major cellular iron exporter) [59].

Consistently, studies in mice with genetic alterations in iron regulatory genes, including inactivating mutations in the gene encoding hepcidin (HAMP), have shown a transient polycythemia [69].

### 2.2. Human Studies

In humans, a number of epidemiological studies have hypothesized a relationship between serum T levels and the erythropoietic process (Table 1). Hemoglobin levels have been related to T status, with lower concentrations in severely hypogonadal men than in those moderately hypogonadal and eugonadal [29].

Therefore, the age-associated decline in T levels has been proposed to account for the decline in erythroid cell mass and the so-called unexplained anemia of older men.

It is very well known that T serum levels decrease by 1% per year, and bioavailable T (free plus albumin bound T) by 2% per year, from the age of 35. As a consequence, approximately 20% of men over 60 years old and 50% of men over 80 years old have serum T concentration below the normal range for young men [101]. Similarly, in women, T levels decrease from the age of 40, approaching prior to menopause the 50% of those present at 30 yr [101].

The most plausible causes of the reduced T levels observed during the aging process include impaired testicular responses to gonadotropin stimuli, coupled with incomplete hypothalamic-pituitary compensation for the fall in total and free T levels [102].

In a recent population-based study, older men with unexplained anemia exhibited lower T levels when compared to their nonanemic counterparts [70]. Interestingly, a higher risk of anemia has been documented not only in men [103] but also in women [27] with low T levels.

Ferrucci and colleagues showed a significant linear relationship between bioavailable T and Hb levels in 905 older participants of both sexes from the “Invecchiare in Chianti” (InCHIANTI) study [27]. Furthermore, low T levels were prospectively associated with an increased risk of anemia after a 3-year follow-up period.

Primary hypogonadism frequently occurs in elderly patients, while secondary hypogonadism is more commonly diagnosed in middle-aged men with multimorbidity including type 2 diabetes mellitus [71, 104, 105]. Grossmann et al. in a cross-sectional cohort study, performed on 464 men with type 2 diabetes, demonstrated that low T levels were independently associated with anemia [106]. Accordingly, Bhatia et al. [107] documented a lower hematocrit together with elevated plasma C-reactive protein (CRP) concentrations in 70 patients with hypogonadotrophic hypogonadism and diabetes. Thus, both low T and chronic inflammation could be identified as risk factors for mild anemia in men with type 2 diabetes [107].

As widely documented, pituitary adenomas are another possible mechanism involved in the development of secondary hypogonadism. In a retrospective review of 197 male patients with pituitary macroadenomas and low T concentrations, Ellegala et al. demonstrated an impairment of hematopoiesis arguing that T levels are independently correlated with hematocrit [72]. It is very well known that the most frequent cause of hypogonadism among the general male population is the androgen deprivation therapy (ADT), commonly used in managing recurrent, locally advanced, and metastatic prostate cancer [73]. Interestingly, cancer patients undergoing ADT are more frequently anemic [108, 109] than those not on ADT, further supporting the hypothesis that T is able to influence Hb levels. Patients with androgen deficiency related to orchiectomy or pharmacologic androgen ablation for prostate cancer typically have a drop of 1 g/dL in Hb levels [110–112]. Interestingly, after a complete hormonal ablation and radiotherapy, this decrease can be seen as high as 2.5 g/dL [7]. Anemia associated with ADT is also a frequent feature in men receiving combined hormone blockade [113]. In this case, the anemia is usually normochromic, normocytic, temporally related to the initiation of androgen blockade, and reversing after combined hormone blockade discontinuation [113].

Finally, the association between T and decline in hepcidin levels in the elderly is still uncertain. In fact, no correlation between hepcidin and T levels was found in a large cohort of patients with unexplained anemia compared to matched, nonanaemic individuals and to patients with known causes of anemia [70].

### 2.3. Intervention Studies

A meta-analysis of 51 intervention studies using different preparations of T showed a significant increase in both Hb (weighted mean difference (WMD): 0.80 g/dL; 95% CI: 0.45–1.14) and hematocrit (WMD: 3.18%; 95% CI: 1.35–5.01) [114]. In particular, T administration proved effective in increasing hematocrit in older hypogonadal males (Table 1). In a preliminary alternate-case controlled trial, older hypogonadal males (mean age 77.6 ± 2.3) with bioavailable testosterone levels less than 70 ng/dL, receiving testosterone enanthate (200 mg/mL) intramuscularly every 2 weeks for 3 months, showed significantly increased levels of total and bioavailable testosterone concentration and hematocrit [74]. Steidle and colleagues [115], in 406 hypogonadal older men treated with two doses of AA2500 T gel (50 mg/day and 100 mg/day) or T patch (two patches delivering 5 mg T daily), reported increased haemoglobin and haematocrit values after 90 days of treatment, with greater levels in the topical testosterone gel. Interestingly, Snyder et al. showed that T patch treatment is an effective strategy to improve hematocrit, with an effect maintained during the 3-year follow-up period [77].

The effectiveness of other molecular forms and different dosage of injectable T in improving hematopoietic parameters has also been studied. Sih and colleagues showed a significant rise in Hb concentration in men receiving injections of placebo or 200 mg of T cypionate biweekly for 12 months [76]. Jockenhövel et al. underlined that different formulations of T in hypogonadal men had different effects on T levels [75]. In particular, the authors showed that T enanthate (TE) 250 mg for 21 days was able to induce a higher elevation of Hb by 21.1 ± 2.6 g/L and hematocrit by 6.4 ± 0.9%. The linear, dose-dependent increase in Hb levels was also shown by Coviello et al. [116] in healthy young and older men concomitantly receiving a long-acting gonadotropin releasing hormone (GnRH) agonist and graded doses of T enanthate. In this study, older people experienced a greater increment in both Hb concentration and hematocrit but not in serum EPO concentrations when compared to younger men. Conversely, a double-blind placebo-controlled trial evaluating the effects of T patch therapy on bone turnover in 39 men (range 40–77 years) showed that 5 mg/day of T therapy for 6 months led to an increase in Hb level but not to significant changes in hematocrit [79].

In anemic male patients [78], both T and 5-dihydrotestosterone treatment resulted in increased plasma and urinary EPO concentrations. In particular, Alexanian [117] documented that fluoxymesterone treatment in normal and anemic men (dose of 40 mg/m^2^), hypogonadal men, and anemic women (dose of 10 mg/m^2^) drove a 5- to 10-fold greater increase in the urinary concentrations of EPO. Unlike animal models, humans are able to retain the responsiveness to androgens even after nephrectomy, probably because of the presence of extrarenal sources of EPO. However, several studies failed to prove the effectiveness of transdermal androgen therapy in increasing EPO levels in anemia of chronic kidney disease [118] or in healthy older men with serum T concentration <475 ng/dL [119].

In healthy young (aged 19–35 years; *n* = 53) and older men (aged 59–75 years; *n* = 56), the administration of weekly graded doses of T enanthate (25, 50, 125, 300, and 600 mg) over a 20-week period induced a significant increase in hematocrit together with decreased hepcidin levels [61].

However, it should be underlined that erythrocytosis is a typical phenomenon occurring in diseases featured by high T levels [120, 121]. Therefore, the detection of erythrocytosis as the most common adverse event of T replacement therapy both in clinical practice and in T trials [122–124] is highly expected.

Erythrocytosis may decrease organ perfusion and the rise of hematocrit levels has been linked to cardiovascular complications [34]. Based on these evidences, the Endocrine Society Guidelines on Androgen Deficiency Syndromes in Men recommend monitoring haematocrit after 3 months from the initiation of T therapy and annually thereafter [125]. However, the clinical benefits and long-term risks associated with T replacement therapy, particularly in prostate-related and cardiovascular-related adverse events, have not been adequately assessed in large, RCTs involving older men [126]. More recently, Snyder and colleagues [127] have described a coordinated set of seven ongoing clinical trials, designed with the aim of determining the efficacy of T treatment in older men with low T and symptoms or signs of impaired mobility, diminished libido, and reduced vitality on objective measures of age-associated conditions, including anemia. Convincing and definitive data are expected to come when these ongoing T trials will be completed.

In the last five decades, further efforts have been made to outline the potential mechanisms underlying the relationship between androgens and Hb and hematocrit. However, data on this matter are limited and not yet conclusive. Therefore, the usefulness of T therapy in anemic men exhibiting an age-related decline in T levels remains to date a controversial issue.

## 3. Estrogens and Estradiol

Estrogens levels, estradiol (E_2_) and estrone (E_1_), are mainly the products of the aromatase-catalyzed T metabolism in men. Similarly to what has been observed for T and IGF-1, these hormones undergo a progressive decline during the aging process, even with different trajectories compared to women [128]. Several investigations suggest potential erythropoietic properties for E_2_. In particular,* in vitro* and* in vivo* studies have documented that E_2_ inhibits both hepcidin transcription and synthesis [129, 130]. Yeap and coworkers in a cohort of community-dwelling adult men (mean age 50 years) showed that E_2_ levels were positively correlated with Hb [131]. Moreover, E_2_, but not T, was also identified as an independent predictor of Hb concentrations in 918 men aged 70–81 from the MrOS (Osteoporotic Fractures in Men Study) [132]. The potential underlying mechanisms by which E_2_ levels influence erythropoiesis are still unknown. Several lines of evidence however seem to support the hypothesis that E_2 _might modulate the hypoxia-induced EPO expression [133, 134].

## 4. IGF-1 and Anemia

### 4.1. Potential Mechanisms

A potential role for IGF-1 in erythropoiesis has been hypothesized in experimental and animal studies, where IGF-1 was able to positively influence various steps of the hematopoietic process [135]. In particular, these biological activities seem to take place through IGF-1 specific binding with two high-affinity membrane-associated receptors [136], both in precursors and mature erythrocytes [137, 138]. By these mechanisms, IGF-1 contributes to maintaining normal erythropoiesis, granulopoiesis, and lymphopoiesis [139, 140] and may regulate several neoplastic haematopoietic processes [138].* In vitro* studies have shown that IGF-1 directly stimulates the proliferation and differentiation of the late stage of primitive erythroid progenitor cells and/or early erythroid progenitor cells [137, 141–144]. Moreover, circulating erythroid progenitors of hematopoietic cells from patients with polycythemia vera are hypersensitive to IGF-1 treatment [145]. Both IGF-1 and IGF-binding proteins are synthesized and secreted by TC-1 murine bone marrow stromal cells [139].

Experimental studies have also clearly documented an effect of IGF-1 on the expansion of the primitive multipotential CD34+ CD38+ stem cells [146, 147]. It is very well known that CD34+ CD38+ stem cells account for the majority of bone marrow hematopoietic stem cell activity [147]. Therefore, a role for IGF-1 similar to that of hematopoietic cytokines has been hypothesized. Moreover, IGF-1 administration in neonatal [148] or hypophysectomized animals [149] enhances erythropoiesis.

At the molecular level, IGF-1 seems also able to increase the expression of cell-surface transferrin-receptors by determining a redistribution from the intracellular compartment to the cell surface [150]. There is further evidence that some of erythropoietic effects of IGF-1 could also be driven by the interactions existing between IGF-1 and IGF-1 binding proteins (IGFBPs) with transferrin, which is known to be the major iron-carrying protein in serum [151].* In vitro*, IGFBP-3 leads to a suppression of IGFBP-3-mediated cellular proliferation and apoptosis, by interacting with transferrin [151]. IGFBP-3 is the storage protein for IGF-1, able to exert paracrine/autocrine actions on cellular growth via IGF-dependent and IGF-independent mechanisms [151]. Interestingly, several evidence indicate that some of IGF-1 erythropoietic effects could be synergistic with EPO and that IGF-1 might replace EPO as a stimulator of erythropoiesis [138, 152, 153].

In mice with chronic kidney disease-associated anemia, combined subtherapeutic doses of EPO and IGF-1 had a comparable effect to a single therapeutic dose of EPO on Hb concentrations [154]. In hormone-depleted cell culture systems, physiological concentrations of IGF-1 (0.5–1 ng/mL), in the presence of EPO, stimulate erythroid cell growth and differentiation from bone marrow or peripheral blood [155].

In 1989 Brox et al., by analyzing an anephric patient's serum with nearly normal hematocrit and a low-to-normal EPO levels, identified a specific 8-kd EPO-like peptide factor, capable of stimulating late erythropoiesis [156]. Interestingly, Congote et al. argued that this erythroid cell-stimulating agent has to be identified in the human IGF-1 [157]. However, the relationship between IGF-1 and EPO remains still unclear, with evidence yielding conflicting results. In male and female rats during accelerated growth IGF-1, but not EPO, concentrations were linearly correlated with total iron incorporation into red blood cells [158]. Similarly, several lines of experimental evidence failed to report any increase in EPO and erythropoiesis indices after IGF-1 treatment. Kling et al., in suckling rats treated with IGF-1 for 4 days, observed higher Hb levels, red blood cell counts, and hematocrit, but no significant changes in plasma EPO levels or reticulocyte counts or plasma iron and erythrocyte iron incorporation rate [159]. Consistently, short-time IGF-1 administration did not promote EPO secretion and erythropoiesis in rat models of protein malnutrition [160]. Finally, GH and IGF-1 were capable of inhibiting EPO secretion both* in vivo* and* in vitro* [161].

IGF-1 might also influence the hematopoietic process and the development of anemia by modulating inflammation [162]. It has been also demonstrated that chronic inflammation, as a result of the unpaired balance between anti-inflammatory and proinflammatory mediators, could be implicated in the pathogenesis of mild anemia in the elderly [163]. In fact, immune and endocrine systems interplay in maintaining homeostasis. From this point of view, IGF-1 has been shown to play a regulatory action in a variety of immune events [163]. If we assume that IGF-1 plays an anti-inflammatory effect [162], decreased IGF-1 concentrations may account for the increased levels of inflammatory molecules, which are known to negatively affect EPO secretion and red cell precursor survival, ultimately resulting in anemia.

### 4.2. Human Studies

In humans, IGF-1 has been hypothesized as a mediator of anemia under physiological and pathological conditions (Table 2). However, the involvement of IGF-1 in maintaining Hb levels has been particularly investigated only in the pediatric population. It has been estimated that about 30% of erythropoietic process in this age-group is influenced by the effect of IGF-1 [164]. In fact, the iron deficiency anemia is a frequent finding in children exhibiting an impaired GH-IGF-1 axis [165]. A positive correlation between hematocrit and serum IGF-1 levels has been also documented in children with sickle cell anemia [166]. IGF-1 also seems to be a regulator of erythropoiesis in a pediatric population with short stature [167] and GH-deficient adults [168]. Decreased IGF-1 levels have been documented in children with suboptimally treated transfusion-dependent *β*-thalassemia compared to children with transfusion-independent *β*-thalassemia [169]. In these subjects, the correction of anemia was associated with a significant increase in IGF-1 levels [167]. The potential role of IGF-1 in the regulation of human erythropoiesis has also been confirmed by studies performed in patients with Laron syndrome (primary IGF-1 deficiency), where the infusion of IGF-1 significantly raises the red blood cell parameters [146]. Interestingly, IGF-1 seems also involved in the regulation of erythropoiesis in uraemic patients, especially when a severe secondary hyperparathyroidism coexists [84].

The role of IGF-1 in adult and older people affected by anemia of chronic kidney disease (CKD) has been also studied [170]. In such cases, the onset of anemia depends on several factors, including the relative or absolute deficiency in EPO production, the shorter survival of red blood cells, the presence of unknown inhibitors of erythropoiesis in uremic phase, hyperparathyroidism, accumulation of aluminium, and nutritional deficiencies (iron, vitamin B12, and folate). Decreased IGF-1 levels were also observed in adults and older patients affected by diabetes-related CKD (DM-CKD). Compared to those subjects with nondiabetic CKD, patients with DM-CKD experienced anemia at an earlier stage of the disease [171]. Kim and colleagues found significantly lower serum IGF-1 levels and Hb concentrations in a group of DM-CKD subjects than in matched CKD patients without diabetes [85].

Similarly, studies performed in healthy prepubertal and pubertal subjects suggested a positive correlation between Hb concentration and both serum IGF-1 and IGFBP-3 levels [172].

In female adolescents, aged 14 to 17 yr, low IGF-1 concentrations were associated with a greater incidence of iron deficiency anemia [173]. In nondiabetic adult subjects with low levels of IGF-1, Succurro et al. identified suboptimal IGF-1 levels as a contributor to the mild anemia by showing a 2.49-fold increased risk in the group with the lowest IGF-1 quartile [83].

Although the potential role of IGF-1 in maintaining Hb concentrations has been addressed in observational studies including subjects of different ages and diseases, very few studies have been carried out in the elderly. This is surprising, because hyposomatotrophism, characterized by typical gradual decline and alteration in GH secretion pattern and IGF-1 production, is a frequent feature of the aging process [174]. In fact, the daily secretion of GH undergoes a progressive reduction with age, esteemed around 14% per decade after puberty. As a consequence of this physiologic process, both IGF-1 and IGFBPs serum levels tend to decline, so that 30% of nonobese older men are at a IGF-1 level below the lowest serum concentration of 16 nmol/L [175]. In women, a profound reduction in GH levels mainly occurs after menopause [176]. The age-related decline in GH-IGF-1 axis activity is known to be a predisposing factor for several clinical adverse consequences, overall termed as “somatopause” [177]. Based on the existing evidence linking low IGF-1 levels and anemia in other populations, it could be speculated that a reduction in IGF-1 levels with aging might contribute to anemia. Therefore, we believe that the onset of anemia in the elderly should be also considered in the context of somatopause.

Previous investigations in a representative sample of 938 older men and women aged ≥65 years from the InCHIANTI Study showed that IGF-1 levels are independently and positively associated with Hb concentration [80]. In men, but not in women, there is also a negative and independent association between IGF-1 and anemia. Some few additional studies were carried out in older individuals to address the relationship between IGFBP-3 levels and indexes of erythropoiesis. Nilsson-Ehle and colleagues studied a community-based cohort of 297 older subjects aged 70 years and showed that IGF-1, but not IGFBP-3, levels influence Hb concentrations independently of EPO levels, sex, and health status [81]. These data are consistent with those of a large study cohort of community-dwelling older individuals from the Aging and Longevity in the Sirente Geographic Area (ilSIRENTE) Study, showing that those with IGFBP-3 concentrations >145.95 nmol/L had a significantly higher mean Hb concentration compared with those in the lower IGFBP-3 level group (13.4 ± 1.4 g/dL versus 12.9 ± 1.9 g/dL, resp.; *p* = 0.02) [82].

### 4.3. Intervention Studies

Intervention studies testing the effect of GH therapy on indexes of erythropoiesis have been focused on GH-deficient children or adults with CKD. In these contexts, RCTs seem to support the beneficial effect of GH on anemia [178, 179]. However, evidence in healthy community-dwelling older individuals is lacking.

rhGH administration in pediatric patients with idiopathic GH deficiency-related anemia resulted in normalization of Hb concentrations and enhancement of erythroid colony growth [180]. More interestingly, the correction of anemia by blood transfusion in subjects affected by *β*-thalassemia and failure to thrive rapidly leads to an improvement in the GH-mediated IGF-1 and IGFBP-3 secretion [181]. Similarly, short-term continuous subcutaneous human GH administration (rhGH 2 *μ*g/kg body weight per 0.1 mL/h for 72 h) in anemic patients with chronic renal failure was able to ameliorate plasma EPO levels and reticulocyte counts [86]. In particular, IGF-1 seems to play an important role in the regulation of erythropoiesis in patients with end-stage renal disease and erythrocytosis without an increased EPO production [170].

In a small RCT conducted in malnourished dialysis patients, Hb levels significantly increased during GH treatment when compared to control group (Table 2) [88]. This is consistent with what has been reported by Chu et al. in malnourished elderly patients who underwent a 4-week low-dose rhGH administration (0.03 *μ*cg/kg 3 times per week) [87].

By contrast, a double-blind, placebo-controlled study of GH treatment in elderly patients on chronic hemodialysis did not show any effect of GH on Hb levels [89]. However, it should be underlined that the dose of GH highly differed between the two considered studies.

Finally, several trials were designed in order to address the effect of EPO replacement therapy on anemia and IGF-1. However, the effect of EPO therapy on IGF-1 serum level is still debated. In uraemic patients, EPO administration led to an enhancement of the GH response to the growth hormone releasing hormone (GHRH) [182]. In hemodialysis patients, EPO therapy was shown to be able to partly correct perturbations in the GH secretory axis, modulating the serum concentration of IGF-1 and IGF binding protein-3 [183, 184].

## 5. Thyroid Hormones and Anemia

### 5.1. Mechanisms

The relationship between thyroid diseases and erythropoietic dysfunctions has been documented in experimental and human studies [185]. In both hypo- and hyperthyroidism, studies have shown red cell abnormalities [186, 187] as well as negative changes in myeloid cell lines [188]. In particular, there is evidence that thyroid-stimulating hormone (TSH), L-triiodothyronine (T3), and L-thyroxine (T4) might exert a direct role in ensuring normal erythropoiesis [189–191]. However, the potential underlying mechanisms by which thyrotropin and thyroid hormones influence anemia have not been fully elucidated.

TSH could affect hematopoiesis by binding to a functional thyrotropin receptor (TSHR), which is found in both erythrocytes and some extrathyroidal tissues [192]. Interestingly, haematopoietic progenitor cells of hypo- and hyperthyroid patients showed a reduction of their proliferative potential together with negative changes in TSHR and pro- and antiapoptotic genes expression [193].

There is also* in vitro* evidence that both T3 and T4 are implicated in the regulation of hematopoiesis by influencing the erythroid precursor proliferative capacity [194]. In rats with bilateral nephrectomy or subdued to bilateral ureter ligature, T3 and T4 were shown to be able to stimulate bone marrow erythropoiesis, especially at higher plasma levels of free active forms [195]. In particular, T3 has been shown to enhance the release of growth factors from leukocytes and to potentiate the erythroid burst-forming unit (BFU-E) proliferation [195, 196]. It may be involved in the control of growth and apoptosis of hematopoietic cells and bone marrow tissue [197]. Moreover, T3 seems also to influence the proliferation of erythroid cells together with inhibitory properties on EPO-induced differentiation [198].

Similarly, a role for T4 in the modulation of erythropoiesis has been documented. Sullivan and McDonald showed a direct *β*2-adrenergic receptor-mediated stimulation of red cell precursors by T4 [199].

At the molecular level, the effects of thyroid hormones on erythropoiesis seem to be mediated by the T3 binding to specific nuclear receptors, that is, TR*α* and TR*β*, which are encoded on separate genes [189]. The endogenous receptor alpha (c-erbA/TR*α*) and the closely related retinoic acid receptor *α* (RAR*α*) have been shown to play a role in the regulation of normal erythroid differentiation [189]. Interestingly, an alteration in c-erbA/TR*α* content seems to negatively affect the balance between erythroid proliferation and differentiation in chicken erythroblasts [200]. Moreover, a mutated form of c-erbA/TR*α* receptor (namely, v-erbA) was responsible for the development of avian erythroleukemias in animals [201]. These data are also supported by studies in TR*α* knockout mouse, where negative changes of fetal and adult erythropoiesis, with reduced numbers of erythrocyte progenitor cells and an impaired erythroid maturation, occurred [202, 203].

In hypothyroid conditions, reduced EPO levels are the most important determinant of anemia. It is very well known that thyroid hormones exert a stimulatory action on the 2-3-diphosphoglycerate concentrations, which is responsible for the delivery of oxygen to the tissues [185, 186]. Interestingly, both lower plasma EPO levels and bone marrow repression have been found in hypothyroid anemia [185]. In human hepatoma cell line, T3 and T4 were able to stimulate erythropoiesis by hypoxia-induced EPO formation in the kidney in a dose-dependent fashion [204]. Thyroid hormones have been proposed to influence EPO gene expression via the induction of hypoxia-inducible factor-1 alpha (HIF-1*α*) synthesis. According to this molecular mechanism, thyroid hormones might act in concert with hypoxic accumulation and activation of HIF-1*α* [205]. However, several lines of evidence strongly argued against the hypothesis that anemia of hypothyroidism is to be exclusively attributable to insufficient EPO levels. In fact, notwithstanding the frequent detection of a normocytic normochromic anemia in hypothyroid patients related to the impaired red blood cell mass and the erythroid progenitors hypoproliferation [206], a normal erythrocyte life span can be observed [185].

The reduced iron utilization [207], the ineffective erythropoiesis and, in long-standing severe hyperthyroidism, malnutrition [208] have been proposed as additional causal factors leading to anemia of thyroid disorders. A recent review indicates that thyroid diseases, hypothyroidism, and hyperthyroidism (overt and subclinical) might cause anemia by modifying the coagulation-fibrinolytic balance [209]. The potential relationship between thyroid hormones and iron status/metabolism has been documented as synergistic and bidirectional. There is evidence that thyroid hormones might increase iron absorption and iron incorporation into erythrocytes [210], with studies showing increased TSH and lower serum T3 and T4 levels [211] in iron-deficient subjects. Conversely, there is evidence that iron deficiency might negatively affect thyroid metabolism by decreasing oxygen transport (impairing nuclear T3 binding and thyroid peroxidase activity) [212].

### 5.2. Human Studies

#### 5.2.1. Observational Studies

A large body of observational evidence suggests an association between thyroid hormones and anemia (Table 3). Overt thyroid diseases have been frequently associated with erythrocyte abnormalities. In particular, pernicious anemia is a frequent finding of hyperthyroidism and thyroiditis, diagnosed in as much as 20–60% of affected patients [213]. Anemia of hypothyroidism is more frequently normochromic normocytic, hypochromic microcytic, and macrocytic, with a severity that varies according to hypothyroidism degree [213]. In contrast, hyperthyroid individuals quite commonly exhibit a concomitant increase in red cell plasma volume and erythrocytosis [207, 214]. Notwithstanding these patients usually exhibit circulating hemoglobin concentrations within the normal range. Interestingly, morphological features of erythrocytes are similar to that of hypothyroidism [214]. In 1975, Fein and Rivlin found a strong association between Graves' disease and the risk of anemia [185]. Subsequently, Horton and colleagues [215] observed lower red blood cells number in the peripheral blood of patients who underwent thyroidectomy. In hyperthyroid patients, Kawa et al. [193] showed higher red blood cells counts, Hb concentration, and hematocrit whereas lower erythrocyte count and Hb concentrations were found in those who are hypothyroid. A retrospective analysis estimated that anemia is a frequent finding in thyroid disease patients, occurring in up to 40.9% and 57.1% of those hyperthyroid and hypothyroid individuals, respectively [216]. Moreover, the authors showed lower mean corpuscular hemoglobin (MCH) and mean corpuscular hemoglobin concentration (MCHC) and higher MCV in hypothyroid individuals [216]. There are several investigations supporting a tight relationship between alterations in bone marrow function and immunological thyroid disorders. A retrospective analysis involving 388 patients with immunological and nonimmunological thyroid disorders (age range 14–89 years) undergoing thyroidectomy showed lower white and red blood cells values, as well as hemoglobin concentrations in the immunological group [217]. Lima et al. by describing four cases of severe pancytopenia in a cohort of patients with thyrotoxicosis by Graves' disease, concluded that thyroid evaluation should be performed in those with pancytopenia even in absence of specific thyroid symptoms [188].

Additional evidence also supports a significant association between thyroid hormones and hematological parameters in euthyroid subjects. Bremner and coworkers in a population-based cohort of 1011 euthyroid older individuals showed that free T4 and free T3 levels, but not TSH, were positively and linearly associated with Hb concentration, erythrocyte count, and hematocrit [90]. However, no adjustment for potential confounders including nutritional parameters (vitamin B12, folic acid, and iron) and renal function was applied. Similarly, Schindhelm and colleagues in a study population cohort of 708 euthyroid subjects (aged 55–85) from the Longitudinal Aging Study Amsterdam demonstrated that free T4 was significantly associated with hemoglobin, hematocrit, and erythrocyte count, whereas TSH was not [91]. The relationship between TSH, free T4, and erythrocyte parameters was further assessed by Lippi et al. in a retrospective investigation in 1050 euthyroid outpatients aged 50 years or older [92]. In spite of the lack of association between TSH, FT4, and Hb or hematocrit, the authors found a significant and positive association between the red blood cell distribution width (RDW) values and thyroid hormone concentrations. FT4 has been also positively correlated with the number of red blood cells and Hb concentration and inversely correlated with MCV and MCH [216].

The association of free T4 with erythrocyte indices, including Hb, red blood cell count, MCV, RDW, and ferritin concentration, has been further assessed in euthyroid adults with or without C282Y homozygosity from the Haemochromatosis and Iron Overload Screening study. In this study, free T4 was positively correlated with Hb concentration and negatively correlated with RDW in those individuals without C282Y homozygosity [218].

Finally, the association between FT4 and anemia was analyzed in relation to ethanol consumption, given the known relationship existing between alcoholism and thyroid function. In a study cohort of 843 euthyroid men aged 30–89 years from the Nagasaki islands study, a significant inverse association between FT4 and risk of anemia was found only in those who did not consume ethanol [93]. Drinkers had also significantly higher Hb concentrations and lower TSH levels than nondrinkers.

However, in spite of the evidence supporting the relationship between anemia and overt thyroid disease, there is a limited number of studies targeting subclinical thyroid conditions, especially in older individuals. This is surprising because subclinical thyroid diseases, defined by circulating concentration of free T4 and free T3 within their respective reference range, in the presence of abnormal circulating concentration of TSH, are more prevalent in the elderly compared to adult or young populations [219]. In this specific age-group, the prevalence of overt and subclinical hypothyroidism is about 20% [220]. The prevalence of hyperthyroidism ranges from 0.5 to 3%, whereas subclinical hyperthyroidism affects 1-2% and 7-8% of subjects in iodine-sufficient and iodine deficient areas, respectively [220, 221]. This is the result of physiological changes in thyroid hormone production, metabolism, and action occurring during the aging process [222], similarly to what has been described for T and IGF-1. In particular, serum TSH and total and free T3 levels progressively decline with age whereas serum total and free T4 levels tend to remain more stable [222]. However, in the elderly, a “low T3 syndrome” is more frequently detected. This term describes a condition with low serum T3 concentrations, abnormal T4 to T3 conversion and higher concentrations of reverse T3 (rT3) (the inactive metabolite of T4) without any obvious sign of thyroid disease [222]. Low T3 syndrome has been associated with a poor health status [222].

In a retrospective cross-sectional analysis conducted in 600 untreated and treated subclinical hypothyroid and primary hypothyroid patients (aged 25–60 years), Bashir and colleagues showed negative changes in different hematological indices (Hb, RBC, MCV, HCT, RBC%, and RDW) only in those untreated individuals with subclinical and primary hypothyroidism [94].

Several lines of evidence apply to the specific setting of subclinical hypothyroidism and iron deficiency anemia. In a case-control study conducted in 57 subclinical hypothyroid women and 61 euthyroid controls, despite the prevalence of iron deficiency, anemia did not significantly differ between the two groups, while mean serum iron and ferritin concentrations were lower in the group with subclinical hypothyroidism [223]. Consistently, Bremner et al. underlined that iron utilization and/or transport might be impaired in subclinical hypothyroid conditions, with lower serum iron concentrations and transferrin saturation in the subclinical hypothyroid compared to the euthyroid [90].

There are also several data supporting the hypothesis that anemia in patients with autoimmune thyroid diseases is more related to a cobalamin deficiency for concomitant autoimmune gastrointestinal diseases [224]. In fact, these group patients exhibit a higher frequency of chronic unexplained anemia (defined as anemia not related to evident or occult bleeding and/or to erythropoietic disorders). It has been demonstrated that anemia of cobalamin deficiency due to an occult chronic atrophic gastritis can occur in multiple autoimmune processes, which include autoimmune thyroid diseases [225]. However, to date, a screening for either B12 and/or folic acid deficiency in hypothyroid subjects or those with subclinical thyroid function is not routinely recommended [95, 213].

Mehmet et al. [213] did not observe any difference in vitamin B12 and folic acid concentrations between subjects with subclinical and overt hypothyroidism, compared to healthy controls. Similarly, Lippi and colleagues, in a retrospective study performed in 946 hyperthyroid or hypothyroid outpatients aged 21–87, reported differences in neither folic acid nor vitamin B12 concentrations [95]. This is consistent with data from Caplan et al. in a study population composed of 56 hypothyroid, 47 hyperthyroid patients, and 103 age- and sex-matched healthy controls [226]. The lack of an association between vitamin B12 and thyroid hormone status was similarly confirmed by Stella et al. [96]. The authors showed that, in a cohort of 279 older adults, levels of serum vitamin B12 were independent of serum TSH quartile distribution [96].

#### 5.2.2. Intervention Studies

There are very few intervention studies that have tested the effects of thyroid hormone replacement therapy on Hb concentrations and erythrocyte parameters (Table 3).

In a study involving 202 hypothyroid patients, levothyroxine treatment normalized anemia by also reducing the degree of anisocytosis in 25% of cases, in the presence of normal vitamin B12, folic acid, and iron concentrations [215]. In young and nonobese untreated subclinical hypothyroid patients, Nekrasova and colleagues [227] documented an increased frequency of anemia that was normalized after starting substitution therapy. Similarly, in 70 Iranian individuals with primary hypothyroidism, three months of levothyroxine administration induced a significant improvement in different hematologic parameters [97]. However, in a randomized, double-blind placebo-controlled trial performed in 63 women (mean age 58.5 ± 1.3 years) with subclinical hypothyroidism, Christ-Crain and colleagues did not show any significant change in Hb concentration and hematocrit after 48 weeks of levothyroxine treatment (85.5 ± 4.3 *μ*g/day), despite demonstrating an increase in EPO levels [98].

Additional evidence suggests that a combined treatment with levothyroxine and iron salts is more effective in inducing an improvement in Hb and ferritin concentrations than levothyroxine or iron salts alone. Data from a recent 3-month RCT including 60 patients with subclinical hypothyroidism and iron deficiency anemia interestingly showed a greater improvement in several hematological parameters in the treatment arm receiving both medications than in the control arm receiving only one of the two [99].

Similarly, Cinemre at al. showed that the T4 coadministration with iron (240 mg/day oral iron plus 75 *μ*g/day levothyroxine), compared to iron in monotherapy (240 mg/day oral), was responsible for a fivefold increase of Hb concentration as well as better blood count indexes [a mean of 0.4 g/dL in the iron group versus 1.9 g/dL in the iron/levothyroxine group] [100]. Interestingly, isolated evidence indicates that iron supplementation may have a beneficial effect on thyroid function, even in iron-deficient patients without iodine deficiency [228].

However, the conclusions of these studies highly differ from those published by Gokdeniz et al. [229] where iron replacement in patients with iron deficiency anemia and normal thyroid function led to a significant decrease in TSH level and to increased free T4 levels.

Therefore, there is very limited evidence suggesting that subclinical and overt thyroid diseases are implicated in the genesis of unexplained anemia. Moreover, there is much uncertainty on whether thyroid hormone replacement therapy might be considered an effective strategy to improve anemia.

## 6. The Potential Contribution of the “Multiple Hormonal Dysregulation” to Anemia in Older Persons

Ageing model is more frequently characterized by a simultaneous deficiency in anabolic hormones with “multiple hormonal dysregulation” rather than a single mild hormonal derangement [30].

An imbalanced anabolic-catabolic equilibrium, which favours catabolism, has been hypothesized as the key pathway of the accelerated aging process [230].

There is recent evidence that anabolic hormones do not operate independently of each other and might have synergistic effects [30]. It is very well known that T mainly acts as anabolic agent while GH exerts an anticatabolic effect [31]. IGF-1 can be also considered the cross-road of many stimuli including DHEAS and T [30].

Hagenfeldt and colleagues concluded that androgen therapy is able to increase the level of IGF-1, which may represent an important mediator of T action [231]. Maggio and colleagues showed that age-related decline in anabolic hormone levels is a strong independent predictor of 6-year mortality in older men [33]. Interestingly, multiple hormonal deficiencies represent a more robust biomarker of health status in older persons than a deficiency in a single anabolic hormone [33].

This concept is of paramount importance because a partial failure of a single hormone system might be partially or fully compensated by one or more parallel systems without producing a significant detrimental clinical outcome. This phenomenon might explain why symptoms associated with the single hormonal derangement are often not specific. Thus, clinical and research efforts to understand the hormonal contributors to anemia should account for all the overall anabolic hormonal status.

## 7. Interactions between Anabolic Hormones in the Regulation of Hematopoietic Process

Anabolic hormonal pathways could be linked to each other to some extent in influencing different steps of the erythropoietic process. However, very few studies have investigated the potential interaction between anabolic hormones (such as T, E_2_, sex hormone binding globulin (SHBG) levels, and GH/IGF-1 axis) and erythropoiesis.

The effects that a combined androgen and estrogen administration might have on some indexes of erythropoiesis were evaluated by Rochira and colleagues in two adult men with aromatase deficiency [232]. These authors used a human model of congenital estrogen deficiency to avoid the concomitant presence of a functioning aromatase enzyme, able to convert exogenous androgens to an intact hypothalamic feedback. The authors found that T, but not E_2_ administration, was able to increase Hb concentrations, hematocrit, and red cell count. Thus, it might be assumed that T aromatization to E_2_ is not involved in the stimulation of erythropoiesis [233].

The interrelationship between serum T, SHBG, and E_2_ and hematocrit has been cross-sectionally evaluated in 1273 men aged ≥20 years enrolled in the NHANES III Survey (1988–1991) [234]. In this study, low free T and high SHBG levels were associated with low hematocrit levels, whereas total and free E_2_ levels were directly associated with hematocrit [235]. Nevertheless, in 492 middle-aged and older men, Yeap et al. observed that total and free T, but not SHBG, were significantly associated with Hb levels, even after adjustment for confounders [103].

Some of T activities on erythropoiesis could also be linked to the GH/IGF-1 anabolic pathway [35]. There is a significant evidence that these two hormones stimulate each other's production and potentiate their respective anabolic or anticatabolic effects [30]. In normal [235] and hypogonadal men [231], T administration was able to increase serum IGF-1 levels. Moreover, the combined administration of GH and T resulted in greater anabolic efficacy compared to the single hormone administration [236, 237]. However, despite the observed increase of IGF-1 levels in men receiving ADT for localized prostate cancer, Colloca et al. [238] showed a decline in the RBC count and Hb concentration after ADT treatment. Carlson et al. did not appreciate any significant change in hormonal parameters including prolactin, testosterone, LH, FSH, TSH, free T4, T3, GH, and IGF-1, before and during EPO therapy in a small cohort of older adults with end-stage renal disease under hemodialysis [239]. Finally, there is evidence that hormonal or growth factors, including insulin, IGF-1, IGF-2, EGF, and basic fibroblast growth factor-2 (FGF-2), might influence EPO gene expression via the induction of hypoxia-inducible factor-1 alpha (HIF-1*α*) synthesis [240–243]. According to this molecular mechanism, these factors might act in concert with hypoxic accumulation and activation of HIF-1*α* [244], suggesting a common pathway underlying the hormonal regulation of erythropoiesis.

## 8. Conclusions

In the last five decades, the erythropoietic role of anabolic hormones on anemia has been particularly investigated in a large number of studies. Different molecular mechanisms by which androgens, IGF-1, and thyroid hormones can affect the hematopoietic process have been identified. However, the interaction between different anabolic hormonal pathways and the contribution of multiple hormonal dysregulation to the pathogenesis of the mild anemia of the elderly has been poorly addressed.

A very limited number of epidemiological studies have tested the role of the age-related decline in anabolic hormones as contributing factor of anemia, especially in older individuals. Notwithstanding the fact that RCTs testing the effects of a single hormonal replacement therapy on different hematological parameters yielded more convincing results, these studies have been focused on cohorts with a wide range of age and multimorbidity distribution. Moreover, hematological indexes were not the primary outcomes in all cases.

In particular, Hb levels have been significantly correlated with the severity of androgen deficiency and T status. The RCTs and registries testing the effects of T therapy on hematological parameters have shown that erythrocytosis is the most common adverse event of testosterone replacement therapy. However, the usefulness of T therapy in anemic men exhibiting an age-related hypogonadism remains to date a controversial issue. The balance between long-term clinical benefits and risks of T replacement therapy, especially at prostatic and cardiovascular level, has not been adequately assessed in large, randomized clinical trials involving older men. IGF-1 has been hypothesized as cause of anemia under pathological conditions including a wide range of anemic disorders in observational studies. IGF-1 is a regulator of erythropoiesis in children with short stature, adults with GH deficiency, and those affected by chronic kidney diseases. A positive and significant correlation between Hb concentration and both serum IGF-1 and IGFBP-3 levels has been also observed in few studies conducted on healthy prepubertal and early pubertal subjects, nondiabetic adult subjects, and older individuals. Although intervention studies testing the effect of GH therapy on indexes of erythropoiesis in several diseases seem to support the beneficial effect of GH administration on anemia, there is limited evidence in healthy individuals.

Finally, a large body of observational evidence suggests an association between overt thyroid hormones anemia and erythrocyte abnormalities. However, there is limited evidence targeting subclinical thyroid conditions, which are more frequently observed in the older population.

Very few intervention studies have tested the effects of thyroid hormones replacement therapy on Hb concentrations and erythrocyte parameters, with most of them showing no beneficial effect of levothyroxine treatment.

In conclusion, despite the biological rationale for considering the decline in anabolic hormones and alteration in thyroid hormones as determinants of mild anemia of aging, the usefulness of hormonal replacement therapy for treating anemia remains to date a controversial issue.

## Figures and Tables

**Table 1:** Observational and intervention studies testing the relationship between testosterone and hemoglobin and anemia.

Author reference	Year	Type of survey	Population	Dose/follow-up	Results
Observational					
Waalen et al. [70]	2011	Case-control	72 m 80 w >65 y UA	—	Lower T levels in the UA group compared with nonanemic controls
Dhindsa et al. [71]	2004	Cross-sectional	492 m 30–94 y	—	Both total and free T were positively associated with Hb levels
Ferrucci et al. [27]	2006	Cross-sectional	396 m 509 w >65 y	—	Total and bioavailable T levels were linearly correlated with Hb concentration and risk of anemia
Ellegala et al. [72]	2003	Cross-sectional	464 m mean age 64 y DM	—	Low total and free T levels were independently associated with reduced Hb levels in DM men
Saylor and Smith [73]	2010	Cross-sectional	70 m 24–78 y DM	—	Free T levels were positively associated with hematocrit and negatively associated with CRP levels
Ferrucci et al. [27]	2006	Longitudinal	274 m 337 w >65 y	3 y	Nonanemic subjects in the lowest quartile (low total and bioavailable T levels had a higher risk of anemia)

Intervention					
Morley et al. [74]	1993	Case-control study	8 m mean age 76 y	TE 200 mg/mL im/2 wk 3 months	T therapy increases hematocrit
Jockenhövel et al. [75]	1997	Double-blind RCT	15 m 51–79 y	TC 200 mg im/2 wk 12 months	T therapy increases Hb concentration
Sih et al. [76]	1997	No RCT	18 m 22–78 y	T patch 6 mg/day 3 y	T therapy increases Hb levels and hematocrit
Snyder et al. [77]	2000	Double-blind RCT	406 m 20–80 y	T gel 50–100 mg/day patch 24,4 mg/day 90 days	Both T preparations increase Hb and hematocrit values compared to placebo
Alexanian [78]	1966	Double-blind RCT	39 m 40–77 y	T patch 5 mg/day 6 months	T therapy increases Hb but not hematocrit
Merza et al. [79]	2006	Double-blind RCT	61 m 18–35 y 60 m 60–75 y	Injection leuprolide depot 7.5 mg/month TE 25-50-125-300-600 mg/week 20 weeks	Hemoglobin and hematocrit increased in response to graded doses of T, greater in older than young men

UA: unexplained anemia

DM: type 2 diabetes

CRP: C-reactive protein

TE: testosterone enanthate

IM: intramuscular injection.

**Table 2:** Observational and intervention studies testing the relationship between IGF-1 and hemoglobin and anemia.

Author reference	Year	Type of survey	Population	Dose/follow-up	Results
Observational					
De Vita et al. [80]	2015	Cross-sectional	402 m 536 w >65 y	—	Negative association between IGF-1 and anemia in men and positive between IGF-1 and Hb in both sexes
Nilsson-Ehle et al. [81]	2005	Cross-sectional	302 m 317 w 70 y	—	IGF-1 positive predictor of Hb levels regardless of EPO, health status, and sex
Landi et al. [82]	2007	Cross-sectional	85 m 168 w mean age 85 y	—	Higher IGFBP-3 level is associated with higher Hb concentration among older people
Succurro et al. [83]	2011	Cross-sectional	491 m 548 w mean age 48 y	—	IGF-1 is an independent determinant of Hb; lower IGF-1 is associated with anemia
Ureña et al. [84]	1992	Cross-sectional	17 m and w mean age 46 y HD	—	Positive correlation between IGF-1 and hematocrit, but not with EPO
Kim et al. [85]	2007	Cross-sectional	41 m 36 w 29–86 y	—	IGF-1 is independently associated with Hb level in DM-CKD patients

Intervention					
Sohmiya et al. [86]	1998	No RCT	3 m 5 w 46–83 y CRF	Infusion rhGH 2 *µ*g/kg/day 7 days	GH treatment increases EPO levels and reticulocyte counts in CRF patients
Chu et al. [87]	2001	Double-blind RCT	19 m and w >70 y	Infusion rhGH 0.09 *µ*g/kg/week 4 weeks	Low-dose rhGH improves nutritional status and physical function in elderly with protein-energy malnutrition
Iglesias et al. [88]	1998	Pilot RCT	8 m 9 w 30–78 y HD	rhGH 0.2 IU/kg/day sc 4 weeks	Short-term rhGH administration increases Hb and hematocrit values
Johannsson et al. [89]	1999	Double-blind RCT	14 m 6 w 53–92 y HD	rhGH 0.6 IU/kg/week sc 6 months	rhGH supplementation produces anabolic effects but does not significantly affect Hb levels

CRF: chronic renal failure

DMn: diabetic nephropathy

HD: hemodialysis

SC: subcutaneously

DM-CKD: chronic kidney disease (CKD) from DM.

**Table 3:** Observational and intervention studies testing the relationship between thyroid hormones and hemoglobin and anemia.

Author reference	Year	Type of survey	Population	Follow-up	Results
Observational					
Bremner et al. [90]	2012	Cohort	504 m 507 w mean age 58 y	—	FT4 levels are positively associated with Hb, hematocrit, and erythrocytes
Schindhelm et al. [91]	2013	Cohort	708 m and w mean age 68 y	—	FT4, but not TSH, is associated with Hb, hematocrit, and erythrocytes
Lippi et al. [92]	2014	Retrospective	221 m 829 w 58–73 y	—	TSH and FT4 are associated with RDW
Shimizu et al. [93]	2013	Cross-sectional	843 m 30–89 y	—	FT4 is inversely associated with anemia in nondrinkers
Bashir et al. [94]	2012	Cross-sectional	216 m 384 w 25–60 y	—	Alterations in hematological parameters in untreated subclinical and overt hypothyroid patients
Lippi et al. [95]	2008	Retrospective	331 m 615 w 21–87 y	—	No difference in the prevalence of folic acid and B12 deficiency between hypo- or hyperthyroid subjects
Stella et al. [96]	2007	Cross-sectional	119 m 160 w 60–85 y	—	Thyroid hormones are associated with vitamin B12 levels, but not with homocysteine

Intervention					
Kazemi-Jahromi et al. [97]	2010	No RCT	14 m 56 w 18–75 y	LT4 101.7 ± 38.3 *µ*g/day 3 months	LT4 supplementation improves hypothyroidism and anemia
Christ-Crain et al. [98]	2003	Double-blind RCT	63 w 18–75 y	LT4 85.5 ± 4.3 *µ*g/day 48 weeks	LT4 replacement significantly increases serum erythropoietin levels but did not affect Hb or hematocrit
Ravanbod et al. [99]	2013	Double-blind RCT	30 m 30 w mean age 32-33 y	Iron 65 mg/day, T4 50 *µ*g/day or iron + T4 65 mg + 50 *µ*g/day 3 months	Higher efficacy of a LT4 plus iron salts in hematological parameters
Cinemre et al. [100]	2009	Double-blind RCT	44 m 6 w 27–55 y	Iron 240 mg/day or Iron + LT4 240 mg + 75 *µ*g/day 3 months	Higher efficacy of LT4 plus iron salts in iron status and blood count indices

RDW: red blood cell distribution width.
